# Automatic and Controlled Processing: Implications for Eating Behavior

**DOI:** 10.3390/nu12041097

**Published:** 2020-04-15

**Authors:** Sophia Fürtjes, Joseph A. King, Caspar Goeke, Maria Seidel, Thomas Goschke, Annette Horstmann, Stefan Ehrlich

**Affiliations:** 1 Division of Psychological and Social Medicine and Developmental Neuroscience, Faculty of Medicine, Technische Universität Dresden, 01069 Dresden, Germany; sophia.fuertjes@uniklinikum-dresden.de (S.F.); joseph.king@uniklinikum-dresden.de (J.A.K.); maria.seidel@uniklinikum-dresden.de (M.S.); 2 Institute of Cognitive Science, University of Osnabrück, 49074 Osnabrück, Germany; cgoeke@uni-osnabrueck.de; 3 Department of Psychology, Technische Universität Dresden, 01069 Dresden, Germany; thomas.goschke@tu-dresden.de; 4 Department of Psychology, Faculty of Medicine, University of Helsinki, 00100 Helsinki, Finland; horstmann@cbs.mpg.de; 5 Leipzig University Medical Center, Universität Leipzig, 04109 Leipzig, Germany

**Keywords:** eating behavior, automaticity, habit, self-control, cognitive control, context-specific proportion congruent

## Abstract

It is a widely held view that humans have control over their food choices and consumption. However, research also suggests that eating behavior is often triggered by contextual cues and guided by automaticities and habits. Interestingly, the dichotomy between automatic and controlled processing has recently been challenged, suggesting that they may be intertwined. In a large female sample (*n* = 567), we investigated the hypothesis that task-based and self-reported measures of automatic and controlled processing would interact and impact self-reported eating behavior. Results analyzed via structural equation modeling suggest that automatic, but not controlled processing, during a modified flanker task, including a context-specific proportion congruent (CSPC) manipulation, was inversely associated with self-reported self-control. The influence of self-control on unhealthy eating behavior (i.e., uncontrolled and emotional eating, heightened consumption of fat and sugar) was only indirect via habitual behavior, which itself had a strong direct impact. Unhealthy eating was further associated with real-life outcomes (e.g., body mass index (BMI)). Our findings suggest that eating behavior may indeed be guided primarily by automaticities and habits, whereas self-control might facilitate this association. Having self-control over eating might therefore be most effective by avoiding contextual cues eliciting undesired automatic behavior and establishing habits that serve long-term goals.

## 1. Introduction

In times where food consumption is not a mere necessity for survival, but rather a topic of lifestyle and personal identity, research on eating behavior has gained much attention. While most of us would like to believe that we deliberately choose the food we eat, it has been suggested that eating behavior may often be more “automatic” than consciously controlled [1,2]. Studies assessing food consumption in laboratory settings have shown that eating behavior can be independent from personal dietary goals [3]. For example, chocolate consumption has been associated with automatic, stimulus-driven behavior during classic reaction-time tasks, but not with snacking intentions [3,4]. Corresponding findings from studies employing real-time diaries of food intake in daily life suggest that eating behavior is largely guided by situational cues instead of intention [5]. Similarly, eating behavior has been found to be guided by habits (defined as a learned response that is activated by context in a “bottom-up” fashion and independent from short-term goals [6]), such as customarily getting a pastry from the bakery on the way to work each morning [7,8,9]. In this context, not only behavioral routines (longer sequences of actions habitually executed in the same contexts), but especially automaticities (an aspect of habit that is effortless, fast, and inflexible [6,10]) appear to play an important role in priming eating behavior by the environment, e.g., reflexively putting ketchup on french fries [11]. It has been suggested that this may be a factor which has contributed to the worldwide increase in the prevalence of obesity [12,13].

Although the role of habits in eating behavior is well-established [1,14], the influence of “top-down” executive control processes, including self-control, also needs to be taken into consideration. For example, individuals high in self-control (which can be conceptualized as the ability to override or change responses, including the goal-directed inhibition of undesired behaviors [15,16]) have been found to experience less conflict during choices between healthy and unhealthy foods [17,18] and to be more likely to maintain successful weight loss [19]. Similar findings have been reported for individual differences in cognitive control (an umbrella term referring to multiple cognitive processes which orchestrate behavior in a goal-directed manner [20]). For example, elevated cognitive control, including superior inhibition of prepotent responses or resolution of “conflict” incited by irrelevant distractors (e.g., in a Stroop task), has been associated with succumbing to less food temptations in real life [21]. Deficient cognitive control, on the other hand, has been linked with heightened snack consumption in laboratory settings and with unhealthy eating behavior in general [22,23]. It has also been found to increase the likelihood of binge eating behavior in real-life assessments of participants with eating disorders [24]. However, meta-analytic data suggest that effects of control processes on eating behavior are relatively small compared to effects of automaticity [25]. Nevertheless, the complexity of the impact of cognitive control on eating behavior [26,27,28,29] also needs to be appreciated. It could be argued that while eating behavior might be relatively automatic in general, sufficient control capacities could strengthen the influence of other aspects such as, for example, goals or intentions [8,28,30].

The evidence reviewed above illustrates the involvement of both automatic or habitual processes on eating behavior on the one hand, and controlled processes on the other. Importantly, recent research has challenged the traditional dichotomy between fast, effortless automatic, versus slow, effortful controlled processing [31], and suggested that the two may in fact be inextricably linked [32,33]. For example, laboratory experiments using adaptations of classic selective attention paradigms (e.g., Stroop or Eriksen flanker tasks) have shown that implicit contextual information can modulate performance in a manner suggestive of automatic priming of top-down control. In such tasks, so-called congruency effects (i.e., slower reaction times (RT) and increased error rates on incongruent relative to congruent trials) are generally taken as a measure of the capacity to override interference (or “conflict”) between target and distractor stimulus features, reflecting controlled processing. The magnitude of such effects is significantly reduced or abolished for stimuli presented in contexts (e.g., locations) associated with frequent conflict—even though participants are unaware of any contextual variation in conflict frequency [33,34,35,36]. This “context-specific proportion congruent” (CSPC) effect is thought to capture how strongly individuals rely on environmental information to automatically adjust top-down control in accordance with current contextual demands. Greater CSPC effects are thought to index a greater tendency to “relinquish the reins” and let contextual cues guide control adjustments [33,34]. For efficiency, we will refer to this phenomenon as automatic processing in the remainder of this article.

Regarding the interplay of automatic and controlled processing in determining eating behavior, recent experimental evidence has shown that basic food attributes (tastiness) are processed earlier (and, arguably, more “automatically”) than abstract attributes (healthfulness), and that this might explain a significant portion of individual differences in eating-related self-control capacity [37]. The literature discussed above also shows that there is involvement of both automatic and controlled processes in eating behavior and that these processes are most likely intertwined [21,26,28]. However, the question of how these two aspects interact regarding eating behavior has not been explicitly addressed in the research to date.

Against this background, we hypothesized that while a generally elevated amount of cognitive control might be associated with more self-controlled behavior, including more controlled eating behavior (as suggested by the literature discussed above [21,22]), the reverse might be the case for automatic processing (gauged by the CSPC effect), as implied by the general association between automaticity and eating behavior [4,5]. More specifically, in the current study, we aimed to test the prediction that individuals who show more pronounced automatic processing during performance of an established CSPC task would exhibit more self-reported automatic behavioral tendencies, which in turn impact eating behavior. Our overarching aim was to answer the question of whether eating behavior is primarily guided by automatic behavioral tendencies rather than by controlled processes, and whether this influence can be captured not only by self-report data but also by an experimental index of automatic processing. We investigated these associations in a large female population-based sample. By employing an online study design, we utilized the advantages of crowdsourcing [38,39], which reaches the general population and allows for efficient collection of large amounts of data.

## 2. Materials and Methods

### 2.1. Participants

602 volunteers between the ages of 18 and 45 were recruited to participate in an online experiment via the crowdsourcing platform Clickworker (“www.clickworker.de” [40]). Given known differences in eating behavior between men and women (e.g., women show more frequent dieting, men consume more meat, etc. [41]) and a higher prevalence of sub-clinical disordered eating behavior as well as eating disorders in women [42], we only recruited women to participate in this study. Better understanding of eating behavior in females could provide an important contribution to further research addressing the question of when and why problematic eating behavior develops into eating disorders. The mean self-reported body mass index (BMI) was 24.48 ± 6.30 and the socio-economic status was distributed normally (calculation according to “Gesundheit Deutschland aktuell” (GEDA) [43]). For more details on sample characteristics, see the Supplementary Materials.

### 2.2. Materials

#### 2.2.1. Conflict Control

**Figure 1:**
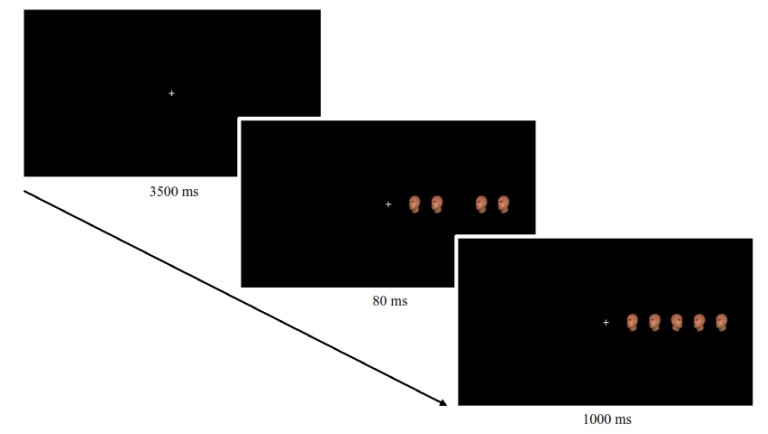
Exemplary trial of the Flanker task. Each unique trial consisted of a fixation cross, followed by the presentation of four flanker faces shown for 80 ms before the identical target face appeared in the center. Participants indicated the viewpoint direction of the target face via keystroke. Half of the 224 trials were congruent (i.e., identical viewpoint direction of flankers and target face), the other half were incongruent (as shown here). Proportion of conflict frequency was manipulated according to context depending on stimulus location: one side of the fixation cross was associated with 75% congruent trials (low conflict condition), the other side with 75% incongruent trials (high conflict condition). Which side of the fixation featured which condition was balanced across participants.

For the experimental assessment of controlled and automatic processing (i.e., conflict control and automatic conflict control adjustments), we employed a modified version of the flanker task with a context-specific proportion congruent (CSPC) manipulation adopted from King et al. [44,45] (see Figure 1). The task was implemented as an online study on the Labvanced platform (www.labvanced.com). Labvanced is an online research tool, which has shown to reliably and precisely record participants’ responses in a wide range of web-based studies [46,47]. In each of the 224 trials, participants had to rapidly and accurately indicate the viewpoint direction (left versus right) of a trial-unique target stimulus face flanked by four distracting stimuli faces. In half of the trials, the target and distractors were congruent, and in the other half, incongruent. Participants were instructed to indicate their response via keystroke. Stimuli randomly appeared either on the left or the right side of a fixation cross. To assess automatic conflict control adjustment, a location-specific manipulation of conflict frequency was employed. One side featured mainly incongruent trials (75% of trials shown on this side), and the other side mainly featured congruent trials (75% of trials shown on that side). Which side was associated with the high versus low conflict context was counterbalanced across the participants. For more details on the apparatus and task procedure, see the Supplementary Materials.

#### 2.2.2. Self-Control and Habitual Behavior

The Brief Self-Control Scale (BSCS) [16] was used to measure two aspects of trait self-control: (low) impulsivity and (high) restraint (Cronbach’s α = 0.50 and 0.70). Habitual behavior was assessed via the two scales of the Creature of Habit Scale (COHS) [48], which assesses automatic responses to situations and behavioral patterns (automaticity scale; Cronbach’s α = 0.83) as well as a tendency to routine behavior (routine scale; Cronbach’s α = 0.85).

#### 2.2.3. Eating Behavior

We used two scales of the short version of the Three Factor Eating Questionnaire (TFEQ-R18) [49] to assess the extent of emotional and uncontrolled eating behavior (Cronbach’s α = 0.87 and 0.89). The third scale of the TFEQ-R18, cognitive restraint, was not used in this study to ensure that eating behavior would be represented in terms of actual behavior (i.e., consumption), not restrictive tendencies. To address nutritional intake as an aspect of eating behavior, we therefore additionally employed the German version of the Dietary Free Fat and Sugar Short Questionnaire (DFS; Cronbach’s α = 0.77) [50,51]. This questionnaire assesses dietary intake of saturated fat and free sugar and has been validated as a self-report instrument reflecting the actual consumption of these nutrients that can be used instead of extensive 24 h food recall instruments [50]. Participants also gave information on whether they followed a specific style of diet (e.g., vegetarianism) and on current and anamnestic weight-loss diets.

### 2.3. Procedure

The study was conducted in accordance with the Declaration of Helsinki, and the protocol was approved by the Ethics Committee of the TU Dresden (135042018). Participants found the study by browsing the clickworker website (www.clickworker.de). The study appeared only for registered female members between the ages of 18 and 45 who lived in Germany. After selecting the study, they were informed about the content and duration of the study, gave consent, and were asked to keep the study displayed on full-screen and eliminate distractions (e.g., turn off mobile phone). Via a customized link, the participants were then routed to the Labvanced web platform, where the study was conducted. In the first part of the study, participants answered questions regarding demographics and filled out the self-report questionnaires. The second part started with detailed instructions of the flanker task and a brief training session (24 trials). Following a short reminder of the instructions, the task ran through until completion. When the task was completed, participants filled out a short questionnaire (as in King et al. [44,45]) to assess awareness of the context-specific congruency frequency manipulation (see Supplementary Materials for details). All participants who successfully completed the study received an individual completion code by the Labvanced experiment software which allowed them to collect their monetary compensation via clickworker.

### 2.4. Data Analysis

#### 2.4.1. Flanker Task Data Analysis

Trials were excluded from all analyses if the response was missing, RT was shorter than 200 ms, or was >/< 3 standard deviations (SD) from the participant’s overall mean, leading to a mean exclusion of 4% of the trials. RT parameters were calculated only on the basis of trials with correct responses. Seven participants whose overall performance (RT and/or error rate) could be considered extreme (>/< 3 SD of the sample mean) were excluded from analyses. Rigorous quality control revealed that several participants did not follow instructions correctly (i.e., used a reversed stimulus-response mapping), which lead to a further exclusion of 28 participants and a final sample-size of 567 participants. For efficiency, we evaluated task performance with a combined measure of speed and accuracy, the linear integrated speed-accuracy score (LISAS [52]; see Supplementary Materials for more details). For each participant, we calculated both the overall flanker effect (LISAS incongruent – LISAS congruent) as a global measure of controlled processing, and the CSPC effect (flanker congruency effect low conflict – flanker congruency effect high conflict) as a measure of automatic processing.

#### 2.4.2. Structural Equation Modeling

**Figure 2:**
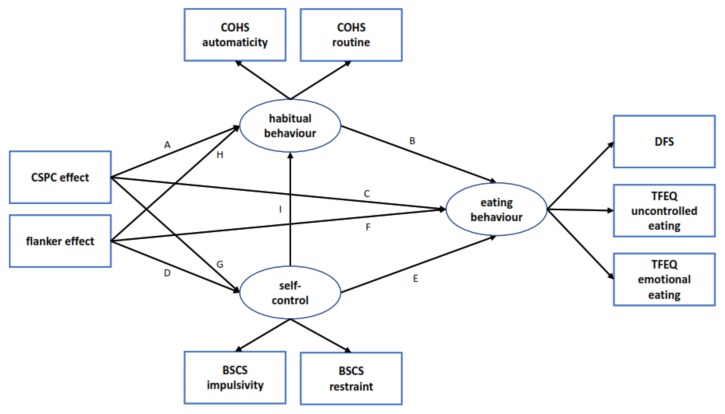
Visual representation of the structural equation modeling (SEM) analyses. Via nested model comparison, a full model (complex model B) was compared to a slightly simpler model (complex model A, paths C and F dropped) and a baseline model (paths C, F, G, H, I dropped). CSPC = context-specific proportion congruent, COHS = Creature of Habits Scale, BSCS = Brief Self-Control Scale, DFS = Dietary Fat and Free Sugar Short Questionnaire, TFEQ = Three Factor Eating Questionnaire.

We employed structural equation modeling (SEM) to investigate associations of task-based measures of conflict control and automatic adjustment of conflict control with self-report measures of self-control, habitual behavior, and eating behavior (see Figure 2). Three latent variables representing eating behavior (estimated from both scales of the TFEQ-R18 and DFS), habitual behavior (estimated from both scales of the COHS), and self-control (estimated from both scales of the BSCS) were built into the model. In a simple baseline model, eating behavior was predicted through self-control and habitual behavior, self-control was predicted through conflict control (flanker congruency effect), and habitual behavior was predicted through automatic conflict control adjustments (CSPC effect). This baseline model was compared to two more complex models via nested model comparison. The first model (model A) additionally included an association between self-control and habitual behavior, between conflict control (flanker congruency effect) and habitual behavior, and between automatic conflict control adjustment (CSPC effect) and self-control. In the second model (model B, also referred to as “full” model), additional direct effects of conflict control (flanker congruency effect) and automatic conflict control adjustment (CSPC effect) on eating behavior were also included (see Figure 2 for a visual representation). Model estimation was conducted using the software AMOS version 21 [53].

To follow up on possible associations with real-life outcomes, we further analyzed the association of eating behavior as measured by the TFEQ-R18 and DFS with self-reported success of weight-loss diets and body mass index (BMI) via logistic and linear regression analyses.

## 3. Results

### 3.1. Sample, Self-Report Data, and Task Performance

The sample demographic characteristics and self-report data are displayed in Table 1. The mean scores of the self-report measures are similar to those reported in previous studies of the general population [16,50,54,55]. The descriptive and inferential statistics of flanker task performance are summarized in Table 2. Mirroring previous findings with the employed task [44,45], the overall flanker congruency effect was significant, and interference was reduced in the high-conflict context relative to the low-conflict context, indicating a significant CSPC effect. Participants’ responses in the post-test questionnaire confirmed the absence of awareness for contextual variation in conflict frequency (see Supplementary Materials Table S2 for more details).

### 3.2. Structural Equation Modeling

**Figure 3:**
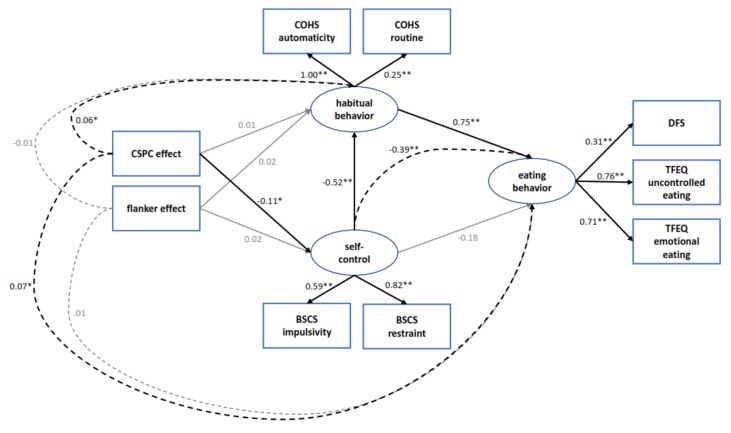
Visual representation of the results of the final SEM model. Values are standardized betas. * = significant at α ≤ 0.05, ** = significant at α ≤ 0.01. Continuous lines represent direct effects, dashed lines represent indirect effects. CSPC = context-specific proportion congruent, COHS = Creature of Habits Scale, BSCS = Brief Self-Control Scale, DFS = Dietary Fat and Free Sugar Short Questionnaire, TFEQ = Three Factor Eating Questionnaire.

The results of the final structural equation model are depicted in Figure 3. All three latent variables could be estimated as expected. As can be derived from the measures of fit in Table 3, the more complex models can be considered a good fit for the data according to generally accepted standards [56]. Given the significant (*p* < 0.01) change in χ^2^, the fit of the two complex models was considerably better than the simple baseline model. The difference of fit between the complex models A and B was nonsignificant. The additional direct effects of model B were nonsignificant and model fit was not improved by adding these effects, therefore the simpler model, model A, was our accepted explanatory model.

The weights in this model show that conflict control per se is not directly related to (self-reported) self-control or habitual behavior, i.e., the overall flanker congruency effect showed no significant associations with these variables (estimated based on the BSCS and COHS). In contrast, greater automatic conflict control adjustment assessed via the CSPC effect was linked to lower self-control and via this association, also indirectly to more habitual behavior. This suggests that a stronger tendency to outsource control to the environment is associated with lower self-reported self-control and more habitual behavior. Self-reported habitual behavior was associated with unhealthy eating behavior (as assessed using the TFEQ and DFS), i.e., more pronounced routine and automatic behavior was linked to more uncontrolled and emotional eating and higher consumption of fat and sugar. Self-reported self-control had no direct association with eating behavior but showed an indirect influence mediated via a shared association with habitual behavior. In line with this, the total estimated impact of habitual behavior on eating behavior (β = 0.75) was stronger than the total effect of self-control (β = −0.57). Mediated via these associations, automatic conflict control adjustment was also significantly associated with eating behavior (as estimated based on the TFEQ and DFS). Specifically, it was indirectly related to more emotional and uncontrolled eating and increased consumption of fat and sugar.

### 3.3. Eating Behavior and Real-Life Outcomes

The results of the additional analyses investigating potential implications for real-life outcomes are summarized in Table 4. Self-reported unhealthy eating behavior, specifically emotional eating, was associated with a higher BMI. For participants who reported having been on a weight-loss diet before (*n* = 392), more unhealthy eating behavior, specifically uncontrolled eating and higher consumption of fat and sugar, was associated with a lower probability of self-reported success in weight-loss diets.

## 4. Discussion

By using self-report as well as experimental indices, the current study addressed the question of whether eating behavior is generally more automatic in nature, triggered by bottom-up environmental cues, or more controlled, guided by endogenous top-down goal representations, and to what extent these processes interact. In a large female sample from diverse socioeconomical backgrounds with a broad distribution of BMI values, participants who tended to let their responses be guided automatically by contextual cues during flanker task performance also reported less self-controlled behavior. Via this connection, such participants also indirectly showed both more automatic and habitual behavior as well as more uncontrolled, emotional, and unhealthy eating. Such eating behavior was in turn associated with a higher BMI. More specifically, individuals who frequently rely on automatic reactions report eating as a reaction to unpleasant emotions, experiencing a lack of control over their eating behavior, and heightened consumption of fat and sugar. In contrast, self-control showed no direct associations with eating behavior, but had an indirect influence via habitual behavior. Together, these results provide novel evidence underlining previous accounts of strong associations between eating behavior and automatic behavioral tendencies [7,9,14]. The results regarding the self-report measures suggest that eating behavior might be largely guided by habits and automatic reactions to contextual cues. In contrast, self-control, as investigated here, seems to have a relatively limited impact. The associations of the self-report instruments with task-based measures of controlled and automatic processing also reflect this pattern. Our findings integrate task-based measures of controlled and automatic processing with self-report data as well as real-life outcomes such as BMI, which offers a novel perspective to previous research.

Our findings suggest that people who rely on contextual cues and automatic behavioral tendencies do so both in cognitive processing as well as in their everyday life, which includes uncontrolled and emotional eating behavior and heightened consumption of fat and sugar. Unhealthy eating behavior could be partly due to contextual cues such as foods high in fat and sugar presented at cash registers or easily available fast food and inviting advertisements. Especially in an environment where unhealthy food is easily available and circumstances invite snacking behavior, the tendency to react automatically when circumstances invite such behavior might make it more difficult for some people to establish or maintain healthy eating behavior. On the other hand, if a specific automatic behavior (which allows for efficient processing) is beneficial, it could yield desirable long-term effects (e.g., following habitual routines in areas such as exercise). Our study complements a line of research highlighting the costs and merits of habitual behavior. While beneficial habits have been found to function as the mediator between self-control and positive life outcomes [25,57,58], we were able to demonstrate possible disadvantages of automatic behavior in the context of eating. Acknowledging that many daily activities are primarily guided by habits and automatic reactions to contextual cues can help to promote a healthy lifestyle. Paradoxically, achieving sufficient self-control might be most efficient via establishing beneficial habits (e.g., a healthy breakfast) that serve long-term goals (e.g., maintaining healthy bodyweight) via pre-commitment. Furthermore, creating situations and contextual surroundings which cue behavior that is in line with those goals (e.g., placing fresh fruit instead of chocolate on your living-room table or changing your daily route to work to avoid the pastry shop) could aid self-control [57]. Studies reporting such moderating associations between habits, self-control, and real-life outcomes (e.g., Reference [59]) are supported by our findings showing that these associations are already present in cognitive processes that exist outside of awareness, which might translate into behavior in everyday life.

The relevance of this interplay between controlled and automatic processes in the context of eating behavior is highlighted by our findings regarding BMI and diets: more unhealthy eating behavior was associated with higher BMI and lower success of weight-loss diets. These findings suggest that the factors found to contribute to unhealthy eating, i.e., habitual and automatic (as opposed to self-controlled) behavioral tendencies, present an obstacle for people who strive to reach or maintain a healthy bodyweight. However, this might also present a starting point for possible solutions. Targeting behavioral automaticities and habits as key factors in weight-loss programs and obesity prevention campaigns might be more effective than fostering self-control. Promising results of habit-based weight-loss programs support this notion [60].

Our findings and interpretations should be considered in the light of some limitations. First, the sample came from the female German-speaking population. Therefore, the results and conclusions may not be generalizable to males or to populations from a different (eating) culture. It should also be noted that the sample included participants who self-reported a history of or current eating disorders (ca. 14% and ca. 5%). Close inspection of the data revealed that these individuals did not cause outliers in the employed measures. Because it was our goal to investigate a sample representative of the general (female) population, we did not exclude these participants from analyses. Second, we did not find any clear associations between task-based cognitive control (as gauged by flanker congruency effects) and self-control or eating behavior, which stands somewhat in contrast to other accounts [61,62]. However, this discrepancy is not entirely surprising considering that associations between cognitive control as captured by laboratory tasks and self-report measures as well as real-life outcomes are generally small and not particularly reliable [63,64,65]. Furthermore, associations between cognitive control and eating behavior are most often reported as moderating or mediating effects [28,29,30]. Third, our findings are limited to the CSPC effect, as observed in our specific task. It has been critiqued that the CSPC effect might not (as generally proposed) reflect automatic adjustment of control, but rather contingency learning [66]. However, our task adheres well to an expert consensus on how to minimize potential confounds in the study of conflict control adjustments [67] and this measure is well-suited to address our research question regarding the complex interplay of automatic and controlled processing. Nevertheless, the findings might not generalize to other related experimental measures [33] or paradigms [68]. Lastly, data regarding BMI and success of weight-loss diets was self-reported. We cannot rule out that participants might have reported their height and bodyweight inaccurately. However, since the data is distributed normally and appears to be representative of the general population, we consider the risk of inaccurate data to be low. The self-reported data on success of weight-loss diets might also not be entirely reliable, and this should be kept in mind when interpreting the associations between eating behavior and success of weight-loss diets.

The present study has shown that reliance on situational cues and automatic tendencies can be measured both via performance during a cognitive laboratory task as well as via self-report. This style of information processing and decision-making influences eating behavior, which was found to be strongly associated with automatic behavioral tendencies (as opposed to self-control). These findings highlight the importance of environmental cues and habits as guiding aspects of eating behavior, which has further consequences for real-life outcomes, such as BMI. People wanting to change their eating behavior might therefore consider approaching this challenge via implementing environmental cues triggering healthy behavior (e.g., fruit instead of sweets placed on the desk) and establishing habits that facilitate the desired behavior (e.g., prepping a healthy lunch to take to work the next day every evening right after dinner). Our findings also support policies aiming to reduce cueing unhealthy food choices through, for example, advertisements or placement of unhealthy items at the top of menus.

## Figures and Tables

**Table 1:** Demographics and questionnaires.

	M	SD	Range
Age	29.25	7.05	18–45
BMI	24.48	6.30	14.9–67.2
BSCS:			
impulsivity	12.05	2.72	4–20
restraint	11.00	3.03	4–20
COHS:			
routine	54.00	10.06	21–81
automaticity	31.03	8.52	11–55
TFEQ-R18:			
uncontrolled eating	19.76	5.84	9–36
emotional eating	6.48	2.75	3–12
DFS	54.67	10.52	29–100

Notes: *N* = 602. M = mean; SD = standard deviation. Age is given in years. BMI = body mass index. BSCS = Brief Self-Control Scale. COHS = Creature of Habit Scale. TFEQ-R18 = Three Factor Eating Questionnaire–Revised. DFS = Dietary Fat and free Sugar Short Questionnaire. Results for the questionnaires are given as raw values.

**Table 2:** Flanker task: Flanker and the context-specific proportion congruent (CSPC) effect.

	High Conflict Condition		Low Conflict Condition	
	Congruent Trials	Incongruent Trials	Congruent Trials	Incongruent Trials
M ± SD (LISAS)	772.46 ± 132.03	892.20 ± 132.30	764.03 ± 126.98	920.51 ± 148.15
Flanker effect	119.74 ± 98.20		156.47 ± 112.13	
	t = –29.03 **		t = –33.23 **	
CSPC effect	36.73 ± 139.50			
	t = –6.27 **			

Notes: *N* = 567. ** = significant at α ≤ 0.01. *t* = value of dependent t-test. LISAS = linear integrated speed-accuracy score, given as an integration of reaction time in ms and error rate in percentage of false response. The Flanker effect is calculated as the difference between incongruent versus congruent trials. CSPC = context-specific proportion congruent. The CSPC effect is calculated as the difference between the Flanker effect in the high versus low conflict condition.

**Table 3:** Model fit of the SEM analyses.

	Baseline Model	Complex Model A	Complex Model B
df	24	21	19
χ²	178.33	53.24	52.84
χ²/df	7.43	2.54	2.78
CFI	0.85	0.97	0.97
RMSEA	0.11	0.05	0.06
AIC	238.33	119.24	122.84

Notes: df = degrees of freedom, CFI = comparative fit index, RMSEA = root mean square error of approximation, AIC = Akaike information criterion. For χ^2^/df, RMSEA, and AIC, lower values indicate a better fit. For CFI, higher values indicate a better fit.

**Table 4:** Associations between eating behavior, body mass index (BMI), and success of weight-loss diets.

	Success of Weight-Loss Diets:		BMI
	Yes	Sometimes	
TFEQ-R18:			
uncontrolled eating	–0.07 *	0.01	–0.05
emotional eating	–0.07	0.02	0.76 **
DFS	–0.04 *	–0.03 *	0.02

Notes: Results of linear (BMI as outcome) and multinominal logistic (success of weight-loss diets as outcome) regressions. Predictors were always included simultaneously. * = significant at α ≤ 0.05, ** = significant at α ≤ 0.01. *N* = 602, *n* = 392 for the analysis regarding the success of weight-loss diets. BMI = body-mass index, DFS = Dietary Fat and Free Sugar Short Questionnaire, TFEQ-R18 = Three Factor Eating Questionnaire—Revised.

## Supplementary Materials

A Word document containing all supplementary material is available online at https://www.mdpi.com/2072-6643/12/4/1097/s1.
