# Supplementary material for: Automatic and Controlled Processing: Implications for Eating Behavior
Source: Nutrients. 2020 Apr 15;12(4):1097. doi: 10.3390/nu12041097 (PMC7230536; doi:10.3390/nu12041097)
Supplement: Supplementary file 1 [file nutrients-12-01097-s001.pdf]

nutrients

MDPI

Supplementary Material

1. Participants

To be included in the study subjects had to be female, between the ages of 18 and 45, and fluent in the German language. Registered users of the crowdsourcing platform Clickworker who met these criteria found a short description of the study on the website and could then decide to participate. The socioeconomic characteristics of the 602 participants are displayed below in Table S1.

Table S1. Socioeconomic characteristics of the sample.

	M ± SD	Percentage (%)
lower education:		
abitur		80%
degree lower than abitur		20%
higher education:		
no higher education		9%
apprenticeship/college education		30%
currently in college/training		24%
Bachelor's degree		19%
Master's degree		17%
ISCED	3.77 ± 1.15	
occupation:		
unemployed		24%
full-time		30%
part-time/marginally employed		35%
other		6%
employee		60%
manual worker		7%
self-employed/freelance		10%
monthly income	2.552 ± 2.225	
SES	13.51 ± 3.04	

Notes. *N* = 602. Abitur = highest degree in Germany, comparable to a high school degree. ISCED = International Standard Classification of Education; score calculated according to Schroedter, Lechert, & Lüttinger [1]. Monthly income is given in Euro (€). SES = socio-economic status; score calculated according to Lampert, Kroll, Müters, & Stolzenberg [2].

10% of the participants reported currently being in treatment for psychiatric disorder: 47 participants indicated suffering from depression, 17 from an anxiety disorder, 11 from a personality disorder (mainly Borderline), 4 from an eating disorder, 4 from posttraumatic stress disorder, and 49 reported other or combined diagnoses (e.g. depression and anxiety, depression and personality disorder). In a separate question specifically asking for eating disorders, 5% of the participants (28 individuals) reported currently suffering from an eating disorder (while not necessarily being formally diagnosed or in treatment) and 15% (88 individuals) responded with having suffered from an eating disorder in the past. Of these 116 participants, 23% reported suffering or having suffered from Anorexia nervosa, 28% from Bulimia nervosa, 15% from binge eating disorder, and the remaining 34% from an unspecified eating disorder.

When asked about their diet, 19.3% of the participants reported following a specific style of diet: 62 participants were vegetarian, 21 vegan, and 35 followed other specific diets such as e.g. low-carb or paleo. 13.8% of the participants reported currently being on a weight-loss diet, 35% reported never having been on a weight-loss diet, and 9% reported being regularly or constantly on weight-loss diets.

2. General Study Details

The programming, presentation, and recording of the study was carried out with the software of the online experiment platform Labvanced (Scicovery GmbH; Paderborn, Germany). Participants were free to take part in the study at the time and location of their convenience but were asked to

eliminate distracting factors such as noisy surroundings to their best abilities beforehand. A desktop computer or notebook running Microsoft Windows, Apple OS, or Linux was required to start the study. Mobile devices such as tablets were not enabled to run the study. Participants could start the study in a web-browser of their choosing among Chrome (40%), Safari (8%), Firefox (42%), MS Edge (7%), or Opera (3%)—Internet Explorer was excluded due to compatibility problems. Starting the study launched full-screen mode, which participants were instructed to keep activated for the duration of the study. To ensure that participants were paying close attention during the reaction-time task, the number of continuous missing responses was counted and the study was terminated (after a previous warning) if participants gave no response for 10 trials in a row. Experiment sessions which were rejected by this control-check were re-opened for new participants immediately. This fully automated procedure enabled the recording of a high number of valid datasets in a relatively short time.

To assess the commitment of the participants, we used instructional manipulation checks (IMC) [3]. A total of five questions to test attentiveness were distributed across the study, disguised as multiple choice questions mixed in with the assessment of demographics or as items of the questionnaires. The vast majority of the participants was very attentive: 406 of the 602 participants answered every IMC correctly, 155 four out of the five. Only 26 participants (4.3%) gave wrong answers to two of the five questions, further 10 participants (1.7%) to three. Four participants (<1%) only answered one IMC correctly and one participant gave wrong answers to all five IMCs. These results are similar to those in studies on IMC [3–5]. To rule out possible influence of attentiveness on our findings, we investigated associations between number of correct IMCs and all questionnaires, parameters of the flanker task, and age. There were no significant correlations. We also repeated all SEM analyses under exclusion of all individuals with less than three correct IMCs—results did not differ from the reported model.

3. Flanker Task

To ensure understanding of the task, participants had to answer two multiple choice questions (“Which faces are you supposed to focus on?—(a) all; (b) only the one in the middle; (c) only the ones on the sides” and “Which key are you supposed to press for “views to the left”?—(a) T; (b) S; (c) B”) after the instructions, before the pre-run. They were looped back to the instructions until they gave the correct answers. The face stimuli used for the flanker task were generated with FaceGen software (Singular Inversions; Toronto, ON, Canada). We used an equal number of left- and right-looking male and female faces with unique identities form various ethnic groups (448 faces total). The viewpoint angles were 45-50°. The task was presented in full-screen mode with a black backdrop and a white fixation cross. Answers were logged via keystroke. To keep possible spatial stimulus-response conflicts minimal, participants were instructed to use “T” and “B” on a QWERTZ keyboard as response keys. For the same reason the response associated with the two keys was randomly assigned to be spatially compatible (left index finger on “T” for left viewpoint direction) for half of the participants vs. incompatible (left index finger on “T” for right viewpoint direction) for the other half. Which of the two keys should be used for which answer (“left” vs. “right” viewpoint-direction of the target) was balanced across participants within each of the two location-based context-specific proportion congruency (CSPC) conditions (right vs. left side of the screen as high conflict context).

To ensure that participants were unaware of the CSPC manipulation, a short questionnaire was filled out after the experiment. Participants indicated the difficulty of congruent and incongruent trials on a visual analogue scale from 1 (very easy) to 10 (very difficult) and answered the question whether they had noticed a pattern in the frequency of congruent vs. incongruent trials (yes/no). If the answered yes, they were asked to answer more fully via free input. Regardless of whether participants indicated having noticed a pattern, all participants were asked to indicate the frequency of incongruent trials on visual analogue scales from 0 (never) to 100 (always) for left side of the screen, right side of the screen, white target faces, dark-skinned target faces, female target faces, and male target faces.

Results revealed that of the n = 567 participants whose Flanker task data was evaluated, only n = 29 reported having noticed a pattern in the frequency of congruent vs. incongruent trials. They described patterns in the order of trials (e.g. 3 congruent, then 2 incongruent, then 4 congruent etc.), a higher frequency of congruent trials depending on viewpoint direction (e.g. more trials with the target face looking to the right), or a higher frequency of congruent trials depending on skin color (e.g. more trials with a dark-skinned target face). No participant described a pattern depending on the side of the screen. The suspected frequency of incongruent trials for the six variables (right side, left side, white, dark-skinned, female, male) can be found below in Table S2. As can be seen, there was significantly less incongruency suspected for dark-skinned and for female faces, but not for right or left side of the screen. Separate t-tests for the two groups according to manipulation condition (high conflict right vs. high conflict left) were conducted for mean suspected frequency of incongruent trials on the right vs. left side. In both groups there was no significant difference (t = 0.00, p = 1.00 for high conflict right; t = 0.52, p = 0.60 for high conflict left).

Taken together, the results of the post-experiment questionnaire show that participants were unaware of the manipulation, which makes it highly unlikely that performance differences between the two conditions were caused by conscious processes.

Table S2. Results of the post-experiment questionnaire.

	**Right Side**	**Left Side**	**White**	**Dark-Skinned**	**Female**	**Male**
suspected frequency of incongruent trials (M ± SD)	50.94 ± 18.38	50.57 ± 18.88	50.40 ± 19.62	46.03 ± 20.59	48.32 ± 19.40	49.73 ± 19.44
difference to "right side" (t)		0.37	0.59	4.50 **	2.68 **	1.20
difference to "left side" (t)	0.37		0.17	4.44 **	2.28 *	0.81

Notes. n = 567. * = significant at $\alpha \leq 0.05$; ** = significant at $\alpha \leq 0.01$.

4. Self-Report Measures

The Brief Self-Control Scale (BSCS) [6] was evaluated via calculation of sum-scores for two scales: impulsivity (four items reflecting intuitive and spontaneous behavior; e.g. *"I often act without thinking through all the alternatives."*) and restraint (four items reflecting self-discipline and engagement in effortful control; e.g. *"I am good at resisting temptation."*). This structure has been established and validated [7].

For the evaluation of the Creature of Habit Scale (COHS) [8] sum-scores for the two sub-scales automaticity (11 items reflecting the tendency to show automatic reactions and behavioral patterns; e.g. *"Whenever I go into the kitchen, I typically look in the fridge."*) and routine (16 items reflecting the tendency to follow routines and establish regularity in daily life; e.g. *"I always try to get the same seat in places such as on the bus, in the cinema, or in church."*) were calculated.

For the two scales of the short version of the Three Factor Eating Questionnaire (TFEQ-R18) [9] also sum-scores were calculated: uncontrolled eating (9 times reflecting subjective loss of control over eating behavior; e.g. *"When I see a real delicacy, I often get so hungry that I have to eat it right away."*) and emotional eating (3 items reflecting the tendency to eat as a response to (negative) emotional states; e.g. *"When I feel blue, I often overeat."*).

The Dietary Free Fat and Sugar Short Questionnaire (DFS) [10] was evaluated via the sum-score of the 26 items assessing the frequency of dietary intake of specific foods rich in saturated fat and free sugar (e.g. cured meats) over the last 12 months.

Self-reported body mass index (BMI) was assessed via free number input items for current height and weight and calculated as BMI = kg/m^2. Specific style of diet was assessed via two items: a multiple choice question *"Do you follow a specific style of diet (vegetarianism, veganism, lowcarb, paleo…)?"* with the possible answers yes or no and (if applicable) a follow-up question *"Which specific style of diet do you follow?"* with a free text input format. Weight loss diets were assessed via a modified multiple choice item of the TFEQ: *"How often have you been on a weight-loss diet?"* with the answer options 1 to 3 times, 4 to 8 times, 9 to 15 times, more than 15 times, regularly, almost constantly, never. If any answer other than never was chosen, a follow-up question assessed success of weight-loss diets

in a multiple choice format: *"Did those weight-loss diets have the desired success?"* with the answer options yes, no, sometimes.

5. Statistics

To circumvent difficulties in data evaluation due to the speed-accuracy trade-off that is frequently present in reaction-time tasks, performance in the flanker task was evaluated via the linear integrated speed-accuracy scores (LISAS). This score estimates performance by correcting reaction time for error rate in a linear manner with equal weighing of both aspects [11]. This score was calculated through Equation (1) for each participant and each condition (high vs. low conflict) according to Vandierendonck [11]:

$$\text{LISAS} = RT_j + \frac{S_{RT}}{S_{PE}} \times PE_j \quad (1)$$

with: RT_j = mean reaction time in condition j; PE_j = error rate in condition j; S_{RT} = overall standard deviation of reaction time; S_{PE} = overall standard deviation of error rate

References

1. Schroedter, J.H.; Lechert, Y; Lüttinger, P. Die Umsetzung der Bildungsskala {ISCED}-1997 für die Volkszählung 1970, die Mikrozensus- Zusatzerhebung 1971 und die Mikrozensen 1976-2004 (Version 1). *ZUMA-Methodenbericht* **2006**, 12, 1-57.
2. Lampert, T.; Kroll, L.E.; Müters, S.; Stolzenberg, H. Messung des sozioökonomischen Status in der Studie "gesundheit in Deutschland aktuell" (GEDA). B*undesgesundheitsblatt-Gesundheitsforsch.-Gesundheitsschutz* **2013**, *56*, 131–143.
3. Oppenheimer, D.M.; Meyvis, T.; Davidenko, N. Instructional manipulation checks: Detecting satisficing to increase statistical power. *J. Exp. Soc. Psychol.* **2009**, *45*, 867–872.
4. Paolacci, G.; Chandler, J.; Ipeirotis, P.G. Running experiments on Amazon Mechanical Turk. J*udgm. Decis. Mak.* 2010, *5*, 411–419.
5. Hauser, D.J.; Schwarz, N. Attentive Turkers: MTurk participants perform better on online attention checks than do subject pool participants. *Behav. Res. Methods* **2016**, *48*, 400–407.
6. Tangney, J.P.; Baumeister, R.F.; Boone, A.L. High Self-Control Predicts Good Adjustment, Less Pathology, Better Grades, and Interpersonal Success. *J. Pers.* **2004**, 72, 271–324.
7. Maloney, P.W.; Grawitch, M.J.; Barber, L.K. The multi-factor structure of the Brief Self-Control Scale: Discriminant validity of restraint and impulsivity. *J. Res. Pers.* **2012**, *46*, 111–115.
8. Ersche, K.D.; Lim, T.-V.; Ward, L.H.E.; Robbins, T.W.; Stochl, J. Creature of Habit: A self-report measure of habitual routines and automatic tendencies in everyday life. *Pers. Individ. Dif.* **2017**, *116*, 73–85.
9. Karlsson, J.; Persson, L.-O.; Sjöström, L.; Sullivan, M. Psychometric properties and factor structure of the Three-Factor Eating Questionnaire (TFEQ) in obese men and women. Results from the Swedish Obese Subjects (SOS) study. *Int. J. Obes. Relat. Metab. Disord.* **2000**, *24*, 1715–1725.
10. Fromm, S.P.; Horstmann, A. Psychometric Evaluation of the German Version of the Dietary Fat and Free Sugar–Short Questionnaire. *Obes. Facts*, **2019**, *12*, 518-528.
11. Vandierendonck, A. A comparison of methods to combine speed and accuracy measures of performance: A rejoinder on the binning procedure. *Behav. Res. Methods* **2017**, 49, 653–673.

© 2020 by the authors. Licensee MDPI, Basel, Switzerland. This article is an open access article distributed under the terms and conditions of the Creative Commons Attribution (CC BY) license (http://creativecommons.org/licenses/by/4.0/).
